# Microsatellite Typing and Antifungal Susceptibility of *Candida glabrata* Strains Isolated From Patients With *Candida* Vaginitis

**DOI:** 10.3389/fmicb.2019.01678

**Published:** 2019-07-31

**Authors:** Neda Kiasat, Ali Rezaei-Matehkolaei, Ali Zarei Mahmoudabadi

**Affiliations:** ^1^Infectious and Tropical Diseases Research Center, Health Research Institute, Ahvaz Jundishapur University of Medical Sciences, Ahvaz, Iran; ^2^Department of Medical Mycology, School of Medicine, Ahvaz Jundishapur University of Medical Sciences, Ahvaz, Iran

**Keywords:** *Candida glabrata*, vulvovaginal candidiasis, antifungal susceptibility, microsatellite genotyping, Iran

## Abstract

Vulvovaginal candidiasis (VVC) is a yeast infection with a global reach and millions of dollars are spent annually for its diagnosis and treatment. Recently, *Candida glabrata* with different degrees of antifungal resistance has been considered as the second most common cause of vaginal infections. The aim of the present study is to determine the antifungal susceptibility and molecular epidemiology profiles of *C. glabrata* isolates from patients with VVC. Sixty-one *C. glabrata* isolates were examined for antifungal susceptibility using the EUCAST broth microdilution method. Moreover, microsatellite length polymorphism (MLP) was used for typing the *C. glabrata* isolates using six microsatellite markers. Overall, 13, 3.3, and 0% of the isolates were non-wild types to itraconazole, posaconazole, and voriconazole, respectively. Sixty (98.4%) isolates were an intermediate phenotype to fluconazole and only one isolate was fluconazole resistant. Microsatellite length polymorphism with a discriminatory power of 0.964 identified 35 distinct types and 24 singleton genotypes. The assessment of the population genetic structure revealed that the non-wild-type population had a moderate genetic differentiation compared to the wild type population (F_ST_ = 0.1457). It was also found that the most common genotypes were G27 (eight strains), G12 (six strains), and G4 (five strains). We found that eight strains were resistant/a non-wild phenotype to itraconazole. Five out of eight (62.5%) resistant/non-wild phenotype strains correlated to a predominant genotype (GT27) and the rest belonged to GT11 (12.5%), GT29 (12.5%), and GT28 (12.5%). The current study is the first molecular epidemiology study in the southwest of Iran and demonstrates the antifungal susceptibility profiles of *C. glabrata* in it. This study shows a wide range of the genetic diversity of *C. glabrata* (35 different genotypes) from VVC in the southwest of Iran. The majority of the non-wild isolates had a dominant genotype or genotypes related to this dominant genotype (clonal cluster one).

## Introduction

Vaginitis or vulvovaginal candidiasis (VVC) is a yeast infection with a global reach and millions of dollars are annually spent for its diagnosis and treatment (Essel et al., 2014). Extensive research has shown that 70–75% of women have experienced at least one episode of VVC in their lifetime. Furthermore, up to 50% of the cases have had at least one episode of recurrent vulvovaginal candidiasis (RVVC) (Richter et al., 2005; Amouri et al., 2011; Makanjuola et al., 2018). Although VVC is not a life-threatening infection, it can lead to abortion as well as postpartum and systemic infections (Al-Aali, 2015). Generally, the persistence of RVVC and resistance to antifungals are more common in *Candida glabrata* (Makanjuola et al., 2018).

The main cause of VVC and RVVC is *Candida albicans* in more than 80–90% of cases (Amouri et al., 2011; Sardi et al., 2013; Rezaei-Matehkolaei et al., 2016). Throughout the last decades, the occurrence of VVC with the non-*albicans Candida* (NAC) species has mainly been due to *C. glabrata* with different degrees of drug resistance and pathogenicity (Sardi et al., 2013; Silva et al., 2017; Segal and Frenkel, 2018). Other NAC species are *C. krusei, C. parapsilosis, C. tropicalis*, and *C. dubliniensis* (Richter et al., 2005; Amouri et al., 2011; Al-Aali, 2015; Makanjuola et al., 2018). Generally, NAC species are more frequently isolated from patients with asymptomatic infections and are intrinsically resistant or less susceptible to azoles (Richter et al., 2005; Sardi et al., 2013; Makanjuola et al., 2018).

The epidemiological knowledge of local microorganisms is an important factor in understanding public health problems as well as the treatment and prevention of infectious diseases. *In vitro* antifungal susceptibility tests and molecular typing are two major keys in epidemiological studies. Several methods have been used for the identification and typing of *Candida* species including multilocus sequence typing (MLST), matrix assisted laser desorption ionization-time of flight mass spectrometry (MALDI-TOF MS), random amplification of polymorphic DNA (RAPD), pulsed-field gel electrophoresis (PFGE), multilocus enzyme electrophoresis (MLEE), and fingerprinting with complex DNA probes (Dodgson et al., 2003; Dhieb et al., 2015; Amanloo et al., 2017). However, microsatellite analysis based on short tandem repeats (STR’s) has been considered as a rapid and reliable technique with a highly discriminatory power (DP) for typing of *C. glabrata* (Abbes et al., 2012;Hou et al., 2017).

Vulvovaginal candidiasis is one of the most common fungal infections among middle-aged Iranian women (Mahmoudabadi et al., 2010; Rad et al., 2011; Mohammadi-Ghalehbin et al., 2016; Rezaei-Matehkolaei et al., 2016). Several frequencies of the disease have been reported from different provinces in Iran including 62.1, 43.3, 40, 26, and 4.8% from Ilam, Babol, Arak, Hamadan, and Zanjan, respectively (Jamilian et al., 2007; Bahram et al., 2009; Esmaeilzadeh et al., 2009; Mohamadi et al., 2015; Habibipour, 2016).

Considering the above points, this study aims to investigate the genotyping of *C. glabrata* strains isolated from VVC in the southwest of Iran based on microsatellite markers. Moreover, the most common genotypes and their association with azoles susceptibility are also investigated.

## Materials and Methods

### Isolates and Identification

In the present study, 61 clinical *C. glabrata* strains from patients with VVC were collected. Forty-six (75.4%) strains were isolated from the vaginal samples of patients in Ahvaz (from January 2017 to March 2018), five (8.2%) strains were unknown (related to cities around Ahvaz), and 10 (16.4%) strains were isolated from the vaginal samples of patients in Bushehr city. These isolates were initially identified using the morphological and microscopic features including small yeasts with budding cells without hyphae/pseudohyphae on corn meal agar (Difco, United States) with 1% Tween 80 and its pink colored colonies on CHROMagar^TM^ Candida (CHROMagar, Paris, France). Then, the isolates were confirmed by PCR using ITS1/ITS4 primers and the PCR products were subjected to sequence analysis (White et al., 1990). All the sequences were compared to reference sequences in the GenBank (NCBI) database via the nucleotide BLAST^TM^ algorithm (similarity values ≥ 99%). Finally, all the nucleotide sequences were recorded in the GenBank database.

### Microsatellite Analysis

The genomic DNA was extracted from the overnight cultures using a lysis buffer and boiling method, purified by phenol-chloroform-isoamyl alcohol (Sigma-Aldrich, Germany), precipitated with isopropanol (Merck, Germany), and washed with 70% ethanol. The DNA was then dried in room temperature and preserved at -20°C. Microsatellite length polymorphism (MLP) was performed using two separate triplex PCRs ([GLM4, GLM5, and GLM6] and [RPM2, ERG3, and MTI]) (Table 1) (Abbes et al., 2011, 2012).

**Table 1:** PCR amplification conditions and contexts each for each Triplex.

Contents	Triplex 1	Triplex 2
Volume	25 μl	25 μl
Multiplex TEMpase 2x master mix	12.5 μl	12.5 μl
DNA	3 μl	3 μl
dDW	3.5 μl	3.5 μl
Microsatellite markers	GLM4, 5 pmol (Revers, Forward), 2 μl	RPM2, 5 pmol (Revers, Forward), 2 μl
	GLM5, 5 pmol (Revers, Forward), 2 μl	ERG3, 10 pmol (Revers, Forward), 2 μl
	GLM6, 5 pmol (Revers, Forward), 2 μl	MTI, 10 pmol (Revers, Forward), 2 μl
Conditions	Denaturation, at 95°C for 10 min	
	Denaturation, 30 cycles at 95°C for 30S	
	Annealing, 30 cycles at 55°C for 30S	
	Extension, 30 cycles at 72°C for 1 min	
	Final extension, at 72°C for 5 min	

Six polymorphic microsatellite markers including RPM2, ERG3, MTI, GLM4, GLM5, and GLM6 were used for microsatellite typing (Abbes et al., 2011; Hou et al., 2017). Each forward primer was labeled at the 5′-side with one of the FAM, HEX, and TAMRA fluorophores (Table 2). Amplification reactions and their programs were performed as described by Abbes et al. (2012). The PCR products were sent to the Macrogen Company for fragment analysis by capillary electrophoresis on an ABI3730XL DNA Analyzer (Applied Biosystems). The internal control used by the capillary electrophoresis was the Genescan^TM^ 500Liz^®^ size standard. Finally, the lengths of the alleles were measured with the GeneMapper Software 5 (Applied Biosystems).

**Table 2:** Microsatellite markers.

Markers	Primers	Primer sequences
*RPM2*	Forward	FAM 5′ ATCTCCCAACTTCTCGTAGCC
	Reverse	ACTTGAACGACTTGAACGCC
*MTI*	Forward	HEX 5′ CAGCAATAATAGCTTCTGACTATGAC
	Reverse	GACAGGAGCAACCGTTAGGA
*ERG3*	Forward	TAMRA 5′AGTGCGAGTGTATGTAAAGAATG
	Reverse	CGTATACCTTATCTCCGTTCAA
*GLM4*	Forward	FAM 5′ AGTGTTCATTGTCGCCTTC
	Reverse	AATGCAGGCTCACCATTTTC
*GLM5*	Forward	HEX 5′ TGGGGATAGTGGGAACTCAA
	Reverse	CGATGATTTCATGTCCGATG
*GLM6*	Forward	TAMRA 5′ GATGATTCTGCCCGTTAGGA
	Reverse	CCTGAAGTAGGTGCCGAGAG

### Antifungal Susceptibility Assay

The *in vitro* antifungal susceptibility of 61 *C. glabrata* isolates to four azole drugs (fluconazole [32 mg/ml] [Serva, United States], itraconazole [2.5 mg/ml] [Sigma-Aldrich, Germany], posaconazole [1.75 mg/ml] [Sigma-Aldrich, Germany], and voriconazole [1.25 mg/ml] [Sigma-Aldrich, Germany]) was determined using the European Committee on Antimicrobial Susceptibility (EUCAST) broth microdilution method [EUCAST definitive document EDef 7.3.1 revision 2017 EUCAST (2017)]. This method was modified with colorimetric indicator Resazurin (Gharaghani et al., 2016) to read the MIC more easily. The MIC is defined as the lowest concentration of an antifungal drug that inhibits the fungal growth. MIC_50_ and MIC_90_ were determined as the lowest concentrations of the antifungal drug inhibiting 50 and 90% of the yeast growth, respectively. According to the EUCAST antifungal clinical breakpoint (v.9.0 valid from 2018-02-12) and Klotz et al. (2016), the fluconazole MIC ratios of >32, >0.002–32.0, and ≤0.002 μg/ml were interpreted as resistant, intermediate, and susceptible.

There are no clinical breakpoints for itraconazole, posaconazole, and voriconazole of *C. glabrata* isolates in the EUCAST broth microdilution test (BMT) guidelines. Therefore, species-specific epidemiological cut-off values (ECVs) were used for the interpretation of isolates as a wild-type (WT) or a non-WT. The ECV for the non-WT to voriconazole and posaconazole is MIC > 1 μg/ml while for itraconazole it is MIC > 2 μg/ml (Meletiadis et al., 2017). Furthermore, MIC_50_, MIC_90_, and MIC_GM_ _(geometricmean)_ were calculated for each antifungal drug. The MIC_GM_ is an average or mean which shows the central tendency or typical value of a set of MICs. In this study, MIC_GM_ was calculated using online software^1^. *Candida parapsilosis* ATCC 22019 and *Candida krusei* ATCC 6258 were also used as quality control organisms for the antifungal assay.

### Statistical Analysis

The dendrogram and minimum spanning tree (MSTree) algorithms were constructed with BioNumerics^TM^ software (version 7.6, Applied Maths, License period: valid from 11/October/2018 until 10/November/2018; License string: 2KCN-45RP-DND7-47WW-FVHP-UV2M) using a categorical value to define the genetic relatedness between the *C. glabrata* isolates. The DP was calculated based on Simpson’s index of diversity^2^ (Chillemi et al., 2016).

The Wright’s Fixation Index (F_ST_) was measured by the FSTAT software version 2.9.3^3^ for the interpretation of genetic differentiation among the populations of *C. glabrata* isolates. The F_ST_ values of 0.0 - 0.05, 0.05 - 0.15, 0.15 - 0.25 and higher than 0.25 demonstrate little, moderate, great and fullyKindly confirm whether the edit made in lines “441” is fine. genetic differentiations, respectively (Abbes et al., 2015).

## Results

### Demographic Characteristics

Of the 61 VVC patients with *C. glabrata*, 49.2% had a single episode or acute VVC, 37.7% had two or more than two episodes of VVC in the last year (multiple episodes), and 13.1% had an unknown history. The demographic details of the patients are shown in Table 3.

**Table 3:** Demographic characteristics of patients with vulvovaginal candidiasis.

Vaginitis (61)	
Age range	18–56 year
Sample sources	
Ahvaz	46 (75.4%)
Bushehr	10 (16.4%)
Unknown	5 (8.2%)
**Vaginitis type**	
Single episode or acute VVC	30 (49.2%)
Multiple episode	23 (37.7%)
Unknown	8 (13.1%)
Predisposing factors	
Low-dose estrogen (LD) user	15 (65.2)
HIV^+^	5 (21.7)
Pregnant	3 (13.1)
Non	38 (62.3%)

### Identification of the Isolates

The DNA sequencing analysis of 61 isolates was confirmed as *C. glabrata* and recorded on NCBI GenBank (Accession No. LC389224-84).

### Antifungal Susceptibility Testing (AFST)

**FIGURE 1:**
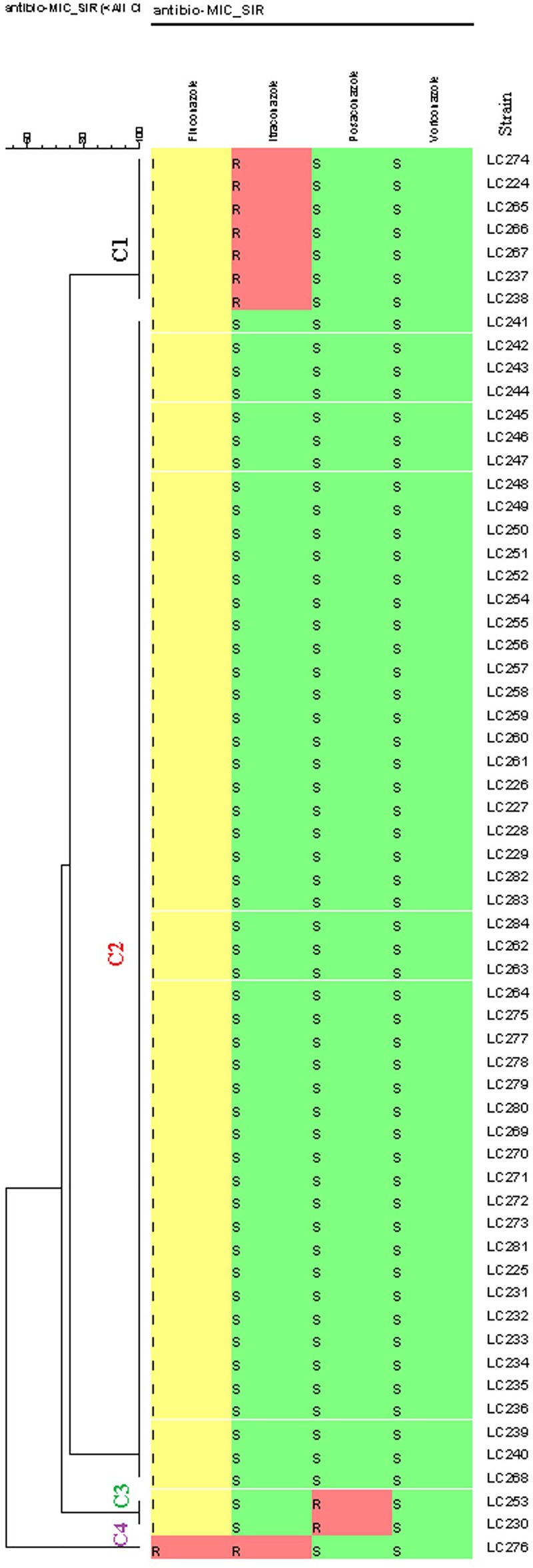
Dendrogram (constructed using the BioNumerics^TM^ software (version 7.6, Applied Maths) of 61 *Candida glabrata* isolates from vulvovaginal candidiasis (VVC) women and the corresponding antifungal susceptibility patterns to the four antifungals. The dendrogram demonstrates four clusters based on their resistance profiles. Epidemiological cut-off values and breakpoints were interpreted according to EUCAST guidelines (2018). A color gradient shows green (WT/sensitive), yellow (intermediate sensitivity), and red (non-WT/resistant) colors.

According to the EUCAST BMT assay and the generated dendrogram, *C. glabrata* isolates were divided into four clusters (C): Cluster 1 (C1) containing seven isolates with a non-WT phenotype to itraconazole, Cluster 2 (C2) containing isolates that were neither a non-WT phenotype nor a resistant phenotype to the tested antifungals, Cluster 3 (C3) containing two isolates with a non- WT phenotype to posaconazole, and Cluster 4 (C4) containing one isolate with a resistant phenotype to fluconazole and a non- WT phenotype to itraconazole. Therefore, only one isolate (1.6%) was cross-resistant/a non-WT to fluconazole and itraconazole in this study (Figure 1).

Sixty (98.4%) of the 61 isolates of *C. glabrata* were intermediate to fluconazole and one (1.6%) was resistant to fluconazole. The rates of non-WT to itraconazole and posaconazole were 13 and 3.3% of the *C. glabrata* isolates, respectively. One hundred percent of *C. glabrata* isolates had WT MICs to voriconazole (Table 4). In general, in this study, 10 (16.4%) of the isolates were resistant/a non-WT to all the tested antifungals. Seven of them were a non-WT to itraconazole, two isolates were a non-WT to posaconazole, and one isolate was both resistant to fluconazole and a non-WT to itraconazole.

**Table 4:** Results of *in vitro* antifungal susceptibility testing of 61 *Candida glabrata* isolates recovered from vaginal candidiasis women by EUCAST BMT.

Species	Antifungals	Minimum inhibitory concentration (MIC) dataset (μg/mL)												MIC Range	MIC_50_	MIC_90_	MIC_GM_	R/non-WT N (%)	CBP_S_/ ECV_S_
		0.0313	0.0625	0.125	0.25	0.5	1	2	4	8	16	32	64						
*Candida*	Fluconazole						1	7	24	23	4	1	1	1-64	4	8	5.56	1 (1.6)	>32
*glabrata* (61)	Itraconazole					**1**	19	33	**7**	**1**				0.5-8	2	4	1.75	8 (13)	2
	Posaconazole	3	2	8	19	24	3	1	1					0.031-4	0.25	0.5	0.3	2 (3.3)	1
	Voriconazole	3	14	19	19	5	1							0.031-1	0.125	0.25	0.14	0	1

*Candida*	Fluconazole						1												
*parapsilosis*	Itraconazole		1																
(ATCC 22019)	Posaconazole		1																
	Voriconazole		1																

*Candida krusei*	Fluconazole											1							
(ATCC 6258)	Itraconazole			1															
	Posaconazole		1																
	Voriconazole			1															

*MIC_*GM*_, MIC geometric mean; R, resistant; Non - WT, non - wild-type; CBP_*S*_, clinical breakpoints; ECV_*s*_, epidemiologic cut-off values.*

### Microsatellite Analysis

**FIGURE 2:**
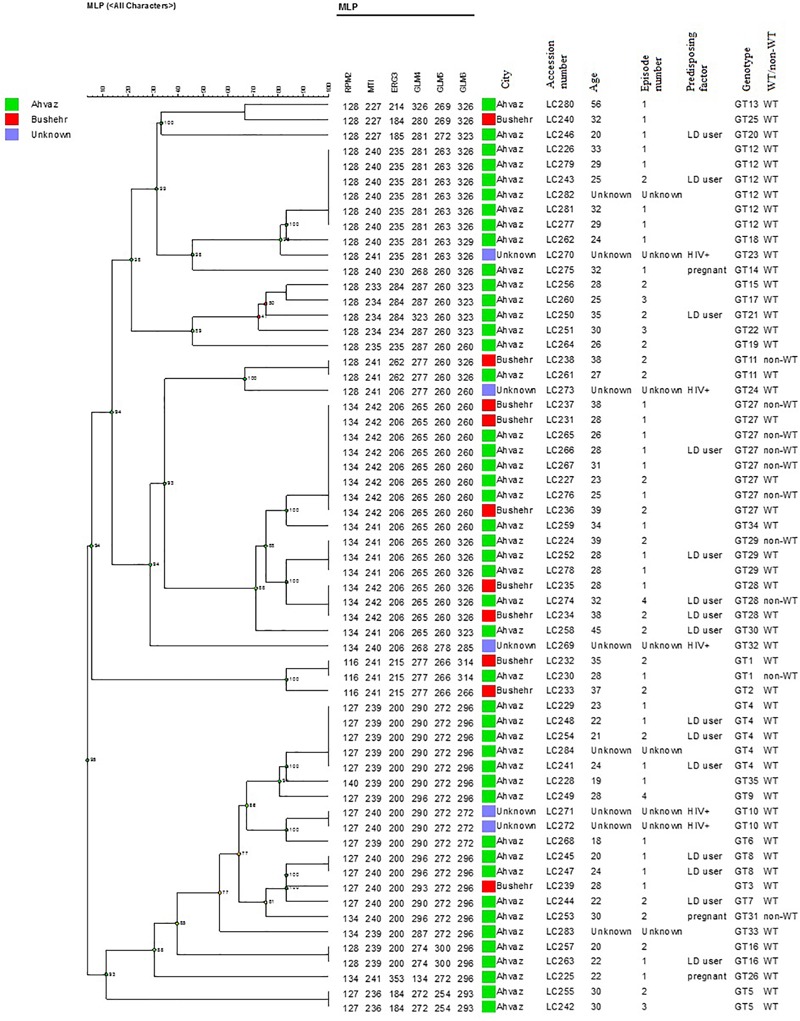
The dendrogram demonstrates the genotyping of 61 vaginal *C. glabrata* isolates collected from the southwest of Iran. The dendrogram was generated using UPGMA based on a categorical analysis of six microsatellite markers by the BioNumerics^TM^ software (version 7.6, Applied Maths).

In the present study, 58 different alleles were identified using six microsatellite loci (RPM2, MTI, ERG3, GLM4, GLM5, and GLM6) in 61 *C. glabrata* isolates. The DP is the average probability that the typing system shows for different types of two unrelated strains randomly sampled in the microbial population of a given taxon. The DP range is from 0 to 1 and the highest DP (DP = 1) indicates that the typing system discriminates between all the isolates. The highest and lowest genotypic diversities were attributed to locus GLM4 (DP_value_ = 0.871) and locus RPM2 (DP_value_ = 0.710) (Table 5). Moreover, 35 different genotypes (DP = 0.964) were obtained from 61 unrelated *C. glabrata* isolates with 24 singleton genotypes (Figure 2). Accordingly, the most frequent genotype was GT27 (eight strains, 13.1%) followed by genotypes GT12 (six strains, 9.8%) and GT4 (five strains, 8.2%). The other remaining genotypes had a frequency of below 5%.

**Table 5:** Features of six microsatellite markers for the 61 *Candida glabrata* isolates from *Candida* vaginitis patients.

Markers	RPM2	MTI	ERG3	GLM4	GLM5	GLM6
Number of alleles	5	9	12	14	8	10
Range size (bp)	116–140	227–241	184–353	134–326	254–300	260–329
Diversity index	0.710	0.827	0.807	0.871	0.733	0.809

### Genotype and Susceptibility to Antifungals

**FIGURE 3:**
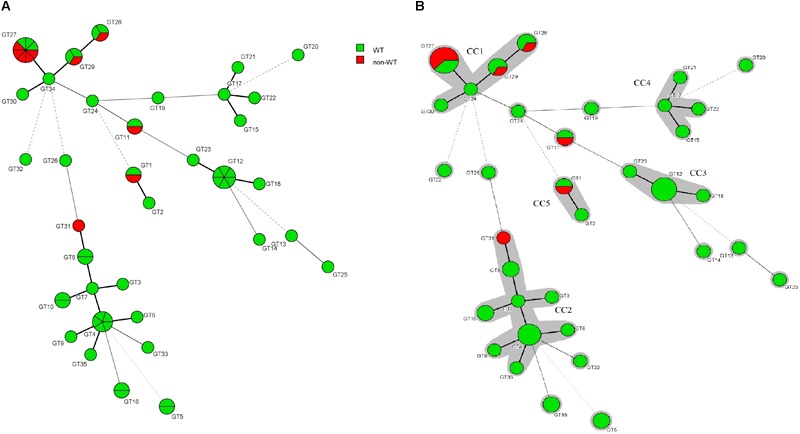
**(A)** Minimum spanning tree created by six loci of microsatellite data of 61 *C. glabrata* isolates by using the BioNumerics^TM^ software (version 7.6, Applied Maths) showing the relationship between the genotypes and susceptibility patterns of four azoles (fluconazole, itraconazole, posaconazole, and voriconazole). Each circle stands for a unique genotype (GT). The green and red circles indicate WT and non-WT phenotypes. The circle size corresponds to the number of isolates belonging to an identical GT from one (the smallest circle) to eight (GT27). Dark, dashed, and thin lines related to one, two, or more different alleles indicate the genetic distance among the GTs, respectively. **(B)** The gray color surrounding some groups of circles represents similar genetic cluster or Clonal Cluster (CC1-CC5). Clonal cluster 1 (CC1) was significantly associated with a non-WT phenotype to itraconazole.

The association between the microsatellite genotype and antifungal drug resistance profile of *C. glabrata* isolates is shown by the minimum spanning tree (MSTree). Based on MSTree (Figure 3), one isolate of the genotype GT1 and the only isolate of genotype GT31 were a non-WT to posaconazole. One isolate of genotypes GT28, GT29, and GT11 and five isolates of genotype GT27 were a non-WT to itraconazole. Also, only one *C. glabrata* isolate was associated with cross-resistant/a non-WT to itraconazole and fluconazole and had genotype GT27. In total, it was found that 50% (5/10) of resistant/non-WT isolates to antifungals belonged to the most common genotype (GT27). In other words, 62.5% (five of eight) of resistant/non-wild phenotype strains to itraconazole correlated to the predominant genotype (GT27) and the rest belonged to GT11 (12.5%), GT29 (12.5%), and GT28 (12.5%). Hence, the majority of resistant *C. glabrata* isolates to antifungal drugs, especially itraconazole, had a dominant genotype, GT27, or genotypes related to this dominant genotype (clonal cluster one).

### Population Genetic Analysis

Wright’s F_ST_ is a measure of the population substructure and is used to analyze the genetic structure differences among populations. F_ST_ value was evaluated between the single-episode and multiple-episode populations (F_ST_ = 0.0053). This finding demonstrated a small genetic structure between two genetic groups of *C. glabrata* isolates. Moreover, F_ST_ was calculated for the non-WT and WT populations (F_ST_ = 0.1457). The wild-type population had a moderate genetic diversity compared to the non-WT population (Table 6).

**Table 6:** Wright’s F_ST_ values for subdivided populations of *Candida glabrata* isolates.

Populations		Isolates (No.)	F_ST_
A	single-episode (*n* = 30) vs. multiple-episode (*n* = 23)	53	0.0053
B	WT (*n* = 51) vs. non-WT (*n* = 10)	61	0.1457

*FST values calculated by FSTAT software version 2.9.3; Population genetic analysis between *Candida glabrata* isolates with different episode (A) and between the wild type and non-wild type phenotype for fluconazole, itraconazole, posaconazole, and voriconazole (B).*

### Association of Iranian Genotypes With Those of Other Countries

**FIGURE 4:**
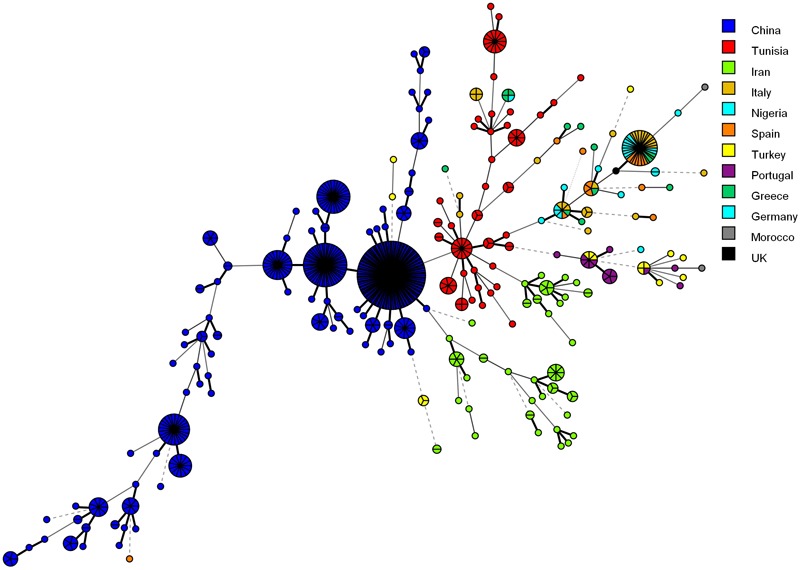
Minimum spanning tree indicating the genotypic diversity of *C. glabrata* isolates from the southwest of Iran compared to those of other countries. Each one of the colored circles indicates one country. Isolates from Iran (*n* = 61), China (*n* = 411), Tunisia (*n* = 85), Italy (*n* = 30), Nigeria (*n* = 17), Spain (*n* = 16), Germany (*n* = 6), Greece (*n* = 15), Morocco (*n* = 2), Portugal (*n* = 15), and Turkey (*n* = 16). Furthermore, a standard strain (CBS2175) from the United Kingdom was used.

Figure 4 shows the MSTree association of the microsatellite genotypes of Iranian *C. glabrata* isolates with those of other countries that used similar microsatellite markers. Based on the MSTree, Iranian genotypes of *C. glabrata* isolates are fully distinct from those in other countries (Abbes et al., 2012; Chillemi et al., 2016; Hou et al., 2017).

## Discussion

Based on the WT defined by the EUCAST method for species-specific *C. glabrata*, only 16.4% (10 of 61) of *C. glabrata* isolates were resistant (r)/had reduced susceptibility (non-WT) to azoles (itraconazole, voriconazole, posaconazole, and fluconazole). In our results, resistance (16.4%) was about 10% more than that obtained by Hou et al. (2017) (6.8%) who tested the isolates with miscellaneous sources. Moreover, by employing EUCAST breakpoints and epidemiological cut-off values, Klotz et al. (2016) mentioned that 25 and 30% of the blood isolates were r/a non-WT to fluconazole and voriconazole, respectively. It seems that the difference among the isolates resistant to azoles can be due to the site of isolation. On the other hand, all the *C. glabrata* isolates were susceptible to voriconazole and only one isolate was resistant to fluconazole. These results are comparable to those of Meletiadis et al. (2017). They demonstrated that the resistance rates to *C. glabrata* were 2.3% for fluconazole and 1.7% for voriconazole. We did not observe any isolate of *C. glabrata* that was susceptible to fluconazole and 98.4 and 1.6% of them were intermediate and resistant to fluconazole, respectively. This trend in reduced susceptibility to fluconazole is a warning for *C. glabrata* isolates, which have acquired a significant resistance to it. Klotz et al. (2016) and Meletiadis et al. (2017) have found that none of the *C. glabrata* isolates were susceptible to fluconazole which was similar to our results.

Several studies have reported high resistance rates of *C. glabrata* isolates to itraconazole with different percentages. Our results revealed that the highest resistance rate (13%) was associated with itraconazole, which is consistent with the studies of Shokohi et al. (2011) (12.5%) and Espinel-Ingroff et al. (2005) (17%). On the other hand, our results are inconsistent with those of Richter et al. (2005) and Amirrajab et al. (2016) who observed a resistance of up to 70% to itraconazole. This difference may be due to the difference in the source of samples and less prescription of antifungal drugs for the treatment of vaginal infections. In other words, resistance to itraconazole correlates with a prior history of the use of itraconazole for prophylaxis and therapy. A lower percentage of resistance to itraconazole (1.2%) was reported by Meletiadis et al. (2017). In our study, the rate of posaconazole resistance (3.3%) was nearly the same as that of Meletiadis et al. (2017) (2.3%).

In this study, we studied the genetic diversity and population structure of *C. glabrata* isolates collected from the southwest of Iran. Notably, all the loci demonstrated a degree of allelic variation from five to 14 alleles for RPM2 and GLM4 markers, respectively. Most of the alleles were common among *C. glabrata* isolates, whereas some of them were less frequent or unique and rare. Finally, our results demonstrated 35 different genotypes of 61 *C. glabrata* strains using six microsatellite markers. In general, although we used the same panel of six microsatellite markers in our study as that used by previous studies, our DP value (0.964) was not consistent with those of Chillemi et al. (2016) (DP = 0.89 for 127 unrelated isolates) and Hou et al. (2017) (DP = 0.88 for 411 unrelated isolates). However, the DP obtained in our study is comparable to that of Abbes et al. (2012) (DP = 0.941 for 85 unrelated isolates) and is higher than the ideal value (0.95) defined by Van Belkum et al. (2007) for genotyping. Therefore, our findings show that a panel of six microsatellite markers could be an excellent typing method for *C. glabrata* isolates recovered from the southwest of Iran.

In our study, the population genetic structure demonstrated a genetic homogeneity (F_ST_ = 0.0053) among *C. glabrata* isolates from two groups of VVC with single and multiple episodes. This finding was inconsistent with the results of Amouri et al. (2012) who found a genetic heterozygosity between two groups of acute and recurrent VVC for *C. glabrata* isolates (Fst: 0.207) as well as, showing a genetic homogeneity between *C. albicans* groups (Fst < 0.05). In agreement with our results, Chong et al. (2003) stated a small genetic diversity not only for *C. glabrata* isolates but also for other *Candida* species such as *C. albicans, C. lusitaniae*, and *C. famata* isolated from two groups (as above). Some researcher also suggested that the occurrence of RVVC could be multifactorial and not necessarily related to specific genotypes (Chong et al., 2003; Richter et al., 2005; Amouri et al., 2011). As was mentioned in the results, most of the detected genotypes (68.6%) were unique. This finding reflects the high genetic diversity of *C. glabrata* isolates from the southwest of Iran and is comparable to the results of previous studies (Abbes et al., 2012, 2015). As is shown in Figure 4, the isolates of other countries did not have identical genotypes in all loci compared to Iranian isolates. This comparison represents a relatively high level of genetic variation of *C. glabrata* isolates and geographical selection (Klotz et al., 2016).

Our findings highlighted a positive association of *C. glabrata* population structures with the predominant genotype (GT27) and r/non-WT to antifungal drugs which is consistent with those of Dhieb et al. (2015). In contrast, other researchers have found no correlation between the predominant genotype or genotypes with antifungal resistance (De Meeûs et al., 2002; Dodgson et al., 2003; Abbes et al., 2011; Klotz et al., 2016; Amanloo et al., 2017). This discrepancy may be due to the differences in geographical locations, the treatment protocols used in different areas, self-medication by patients, sociocultural conditions of people, and other effective factors on drug resistance.

## Conclusion

The current research is the first molecular epidemiology study in the southwest of Iran and demonstrates the antifungal susceptibility profiles of *C. glabrata* in it. This study shows a wide range of the genetic diversity of *C. glabrata* (35 different genotypes) from VVC in the southwest of Iran. The majority of the non-wild isolates had a dominant genotype or genotypes related to this dominant genotype (clonal cluster one).

## Data Availability

The datasets generated for this study can be found in NCBI GenBank, https://www.ncbi.nlm.nih.gov/nuccore/?term=kiasat.

## Ethics Statement

This project was approved by the ethical committee of the Ahvaz Jundishapur University of Medical Sciences (Registered Code: IR.AJUMS.REC.1396.912). Furthermore, all the patients attended midwifery clinics and vaginal sampling was a routine part of their therapy. All the patients have a file in these clinics containing their signed consent forms. In addition, verbal permission was also obtained from the patients.

## Author Contributions

AM and AR-M conceived and designed the manuscript. NK performed the experiment and drafted the manuscript. NK, AM, and AR-M data analyzed and interpreted the results.

## Conflict of Interest Statement

The authors declare that the research was conducted in the absence of any commercial or financial relationships that could be construed as a potential conflict of interest.

## Footnotes

1 http://www.meracalculator.com/math/geometric-mean.php

2 http://insilico.ehu.es/mini_tools/discriminatory_power/index.php

3 http://www2.unil.ch/popgen/softwares/Fstat293dist.exe
